# Incorporating religion and spirituality into the design of community-based physical activity programs for African American women: a qualitative inquiry

**DOI:** 10.1186/s13104-017-2830-3

**Published:** 2017-10-23

**Authors:** Rodney P. Joseph, Barbara E. Ainsworth, LaTanya Mathis, Steven P. Hooker, Colleen Keller

**Affiliations:** 1 Center for Health Promotion and Disease Prevention, College of Nursing and Health Innovation, Arizona State University, 550 N 3rd Street, Phoenix, AZ 85004 USA; 2 Exercise Science and Health Promotion Program, School of Nutrition and Health Promotion, College of Health Solutions, Arizona State University, 550 N 3rd Street, Phoenix, AZ 85004 USA; 3 Community member of the metropolitan Phoenix area, Phoenix, Arizona USA

**Keywords:** Exercise, Physical activity, Black, Women, Religion, Spirituality

## Abstract

**Objective:**

Limited research has examined how aspects of religion and spirituality can be incorporated into community-based physical activity programs delivered outside of religious institutions. The purpose of this study was to qualitatively explore how spirituality and religion can be leveraged in the design of community-based physical activity programs for African American women delivered outside of faith-based or faith-placed settings.

**Results:**

Three focus groups were conducted were conducted with 23 African American women (M age = 37.8 years, M BMI = 39.6 kg m^2^). Results showed that incorporating aspects of spirituality (i.e., words encouraging connectedness to a higher power, meditation, mind–body activities) into a physical activity program was universally accepted among participants, regardless of religious affiliation. In contrast, including concepts of religion (i.e., bible verses and/or quotes from religious leaders) was controversial and not recommended among women who did not identify with a religious faith. Findings indicate that when developing community-based physical activity interventions that will not be delivered through faith-based or faith-placed settings, researchers should avoid references to specific religious beliefs. Instead, interventions should focus on spirituality and emphasize the mind–body relationship between physical activity and an African American women’s inner-being and her connectedness with a higher power.

*Trial Registration* ClinicalTrials.gov NCT02823379. Registered July 1, 2016

## Introduction

African American (AA) women exhibit low physical activity (PA) levels [1] and are disproportionally burdened by associated cardiometabolic disease conditions [2–4], emphasizing the need for effective interventions to promote PA in this population. Religion and spirituality have an extensive history in AA culture [5, 6] and have been the focus of many interventions designed to promote PA [7, 8]. Programs that incorporate religion and spirituality are viewed as advantageous because they acknowledge the deep-rooted religious beliefs and social networks of AA women and leverage them to promote PA [9, 10]. Such programs are generally known as either faith-based or faith-placed. Faith-*based* programs formally incorporate aspects of religion (i.e., scripture/biblical stories) into PA promotion efforts. Faith-*placed* programs are delivered through religious institutions (e.g., churches), but do not necessarily incorporate aspects of religion into formal intervention activities. However, as others have noted [11], faith-placed programs are often augmented by congregation members during delivery to include religious references; which makes them very similar to faith-based programs.

Despite the large body of research on faith-based and faith-placed PA interventions, few studies have examined how religion and spirituality can be incorporated into PA interventions delivered outside of religious institutions. The purpose of this report is to describe how spirituality and religion can be incorporated into PA programs for AA women delivered outside of faith-placed or faith-based settings. Data for this report were collected as part of a larger on-going study that focused on: (1) collecting empirically-driven qualitative data on perceptions, manifestations, and determinants of PA among AA women to improve and refine the cultural relevance of a an established PA program for AA women, and (2) testing the refined intervention in a randomized control trial to increase PA and reduce cardiometabolic disease risk (ClinicalTrials.gov Identifier: NCT02823379).

We conceptualized religion and spirituality as similar, yet distinct concepts. We defined religion as adherence to a formal belief system in which practices are associated with tradition, and where there is agreement among members regarding what is believed and practiced [12]. Moreover, in most Christian religions (which is the predominant religion among AA women [13]), there is shared belief in a supernatural creator (i.e., God) that informs these formal practices and beliefs [12]. We defined spiritually as a more general, unstructured, personalized, and naturally occurring phenomenon where a person seeks closeness and/or connectedness between him/herself and a higher power or purpose [12, 14, 15]. Based on these definitions, both religion and spirituality focus on increasing connectedness with a higher power, but each has a different approach. It also is important to note that these concepts are not necessarily competing belief systems; rather, they can be complementary to each other or independent in their own right [16].

## Main text

### Methods

Data were collected from 23 sedentary and obese AA women. The primary study [17] from which the data were derived included a total sample of 25 AA women. The sample size in this report is smaller than the primary study because two participants did not attend the focus group sessions exploring the topics of religion and spirituality. Based on our previous research experience, the primary study’s a priori sample size of 25 was determined as ideal to provide adequate coverage and depth of the topics explored. Readers are referred to a recently published article [17] for a more in-depth description of the study design, development process of focus group guides, and primary qualitative findings.

Participants were recruited using multiple community-based strategies, including email listservs, newsletters, advertisements on social media, and local websites targeting the AA community. Eligibility for participation included: (a) self-reported as AA, (b) between the ages of 24–49 years, (c) body mass index (BMI) ≥ 30 kg m^2^, and (d) < 60 min/week of moderate-to-vigorous intensity PA according the 2-item Exercise Vital Sign questionnaire, which has been validated for PA assessment among AA women [18]. No further inclusion/exclusion criteria were specified.

Data reported are from three focus group sessions. The number of women attending each focus group session was 9, 8, and 6, respectively. Focus groups were led by an AA woman (LMM) with over 3.5 years of focus group facilitation experience. The P.I. of the study (RPJ) was also present during the focus group sessions to serve as note taker of participants’ non-verbal communications. Topics explored during focus groups were derived from previous PA research with AA women [19–24] and a critical review of the extant literature. The discussion guide used in the focus group sessions is illustrated in Table 1. Of note, participants were not provided with the definitions spirituality or religion during the focus groups. Rather, it was up to them to operationalize these topics through group discussion.

**Table 1:** Focus group guide

Topic targeted	Focus group question
Religion/spirituality	1. Some African American women say that incorporating spirituality or religion into a physical activity program can help motivate or encourage physical activity. How would this influence your desire or motivation for physical activity? Probes: a. How would this *encourage* you to be more physically active? b. How would this *discourage* you to be more physically active?
Religion/spirituality	2. If we were to incorporate religion or spirituality into a physical activity program, how would you like it incorporated? Probes: a. How would you feel about including short bible verses that relate to being physically active? b. How would you feel about having religious figures help deliver physical activity promotion messages? i. Who would these religious figures be? 1. Formal leaders (Pastor, Sunday school teacher) or informal leaders (elders)? c. How would you feel about including inspirational quotes from religious leaders?
General question	3. Is there anything that we have not discussed today or anything else that you would like to share before we end the focus group for the day?

All focus group sessions were audio recorded and transcribed verbatim. Participants were provided an identification number for reporting purposes. Transcripts were imported into NVivo qualitative analysis software (version 11) for analysis. Descriptive content analysis [25] was used to analyze the focus group data. Data were coded using a multi-phase approach, which has been reported elsewhere [17]. Saturation of themes was not formally evaluated by the research team, as we, along with other researchers [26], question its appropriateness in non-grounded theory qualitative research. Likewise, due to the collaborative nature of data analysis, a formal statistic of inter-rater reliability was not calculated. Quantitative analysis of demographic data was completed using SPSS version 23.

### Results

Demographic characteristics of participants are presented in Table 2. Qualitative findings from participants were classified into three overarching themes: (1) Incorporating religion into a PA program was viewed as favorable, (2) Incorporating religion into a PA program was either cautioned or discouraged, and (3) Incorporating spirituality into a PA program: a universally accepted approach. Each of these themes is described in more detail below. Table 3 provides participant quotes supporting each theme.

**Table 2:** Baseline demographic characteristics of participants

Variable	Mean	SD
Age	37.9	7.8
BMI	39.6	7.5
	N	Percent of study sample
Marital status		
Never married	17	72
Married	2	10
Divorced	4	18
Highest level of educational		
Some college	6	26.1
Bachelors’ degree	11	47.8
Master’s degree	4	17.4
Doctoral Degree	2	8.7
Employment status		
Unemployed	0	0
Employed outside of the home	25	100.0
Income level		
Less than $25,000	3	13
$25,001–$50,000	11	47.8
$50,001–$75,000	6	26.1
$75,001–$100,000	1	4.3
$100,000 +	2	8.7
Children in household		
0	13	56.5
1–2	7	30.4
3–4	3	13.0

**Table 3:** Qualitative themes with illustrative participant quotes

Theme	Example quotes
Incorporating religion into a PA program was as favorable	I think it’s encouraging [referring to incorporating religion into a PA program] because it gives you a point of reference… (Focus Group # 1, Participant 19)
	I would say that that would be extremely encouraging for me [referring to incorporating religion]…I would gravitate more towards that because like seeing the Scripture is like it’s a mental focus. And I would be renewing my mind. (Focus Group # 1, Participant 17)
	I would have to say that my spirituality, my faith, has a lot to do with encouraging me to increase my physical activity…reading in the Bible about my body being a temple of the Lord. How I am I to fulfill my purpose on the earth if I’m not healthy to accomplish that? (Focus Group # 1, Participant 1)
	Tie it in with Scripture [referring to how to incorporate religion into a PA program], with the whole your body is your living sacrifice, and you’ve got to keep your temple holy… give God praise. (Focus Group # 1, Participant 6)
	When I was using my prayer time and exercise time together, then I was physically fit and more mentally fit… For me, yes [I would like religion incorporated into a PA program]… (Focus Group # 2, Participant 20)
Incorporating religion into a PA program was either cautioned or discouraged	Each individual is different. So if you have a person who is really dedicated to the Bible versus somebody who takes things with a grain of salt, you’re tiptoeing around. That’s a touchy subject. So that’s something where it depends on the person. (Focus Group # 1, Participant 19)
	This sounds weird but if the church across the street was offering a free workout class, I would go but I don’t want to hear about church….Even though it’s on church grounds, I feel like if I like you then I’ll come to your church, but I don’t want you to push the church on me. (Focus Group # 2, Participant 13)
	“I’m an atheist so that wouldn’t work [in reference to including religious quotes or passages]. That wouldn’t work for me at all.” (Focus Group # 2, Participant 11)
	“To pull people from off the street or whatever and try to throw a class together and then incorporate religion, neh, that might not work as well.” (Focus Group # 2, Participant 13)
	“I don’t see a connection…[referring to incorporating religion into a PA program] (Focus Group #3, Participant 9)
Incorporating spirituality into a PA program: a universally accepted approach	I’m more spiritual than religious now. So for me, maybe having some aspects of the Bible in there would be offensive to me, but I would be receptive to some type of meditation, music or something like that, something relaxing, versus words. Or even words of affirmation, that would be encouraging as well. (Focus Group # 1, Participant 4)
	With spiritually I’m thinking, like I’m meditating or chanting or whatever it is that I’m doing at the time, I feel more self-worth. (Focus Group # 2, Participant 15)
	“I would really just enjoy conversation, spiritual related conversation while working out… There are followers of Christ who I literally do not want anything to do with. Then there are people who maybe don’t have a faith or have a faith completely opposite of what I believe in. They’re so goodhearted. It’s like I just want to soak them up. (Focus Group # 2, Participant 24)
	I think in one of the other meetings [referring to another focus group session] I was talking about what makes me interested are things like it doesn’t have to be religious, per se, but spiritual… It’s like I’m not just counting how many jumping jacks I’m doing every day. I’m breathing, I’m meditating, or thinking about my higher power. (Focus Group # 2, Participant 22)

#### Theme 1: Incorporating religion into a PA program was viewed as favorable

During initial discussions on this topic, several women discussed various religious institutions that offer group-based PA classes to both church members and non-members. Some women reported attending these sessions previously and having positive experiences, while others only reported awareness of their existence. As conversations progressed, numerous women spoke favorably about incorporating religion into a PA program. One participant stated the following when discussing how her religion motivates her to be physically active (Focus Group #1, Participant 17), *“I would have to say that my spirituality, my faith, has a lot to do with encouraging me to increase my physical activity…reading in the Bible about my body being a temple of the Lord. How I am I to fulfill my purpose on the earth if I’m not healthy to accomplish that?”* Participants described various ways in which religion can be incorporated into a PA program, including incorporating scripture into intervention messages and having religious leaders be a part of the program.

#### Theme 2: Incorporating religion into a PA program was either cautioned or discouraged

As conversations on the topic of religion progressed, several participants reported negative experiences they have had while attending church services or organized church events. One participant (Focus Group #1, Participant 3) described the following, *“I haven’t had any positive experiences at church, being there were comments… like, ‘Oh, you’re really heavy.’”* These negative experiences tempered the desire for some women to participate in a PA program that incorporated religious references. Likewise, several women reported that they were not religious and including religious references would be offensive and/or irrelevant to their lives. Collectively, the women reached a consensus that if religion was to be incorporated into a PA intervention, the program should be delivered through a formal religious institution, as it would help alleviate any differences or concerns associated with offending women who were not religious.

#### Theme 3: incorporating spirituality into PA program: a universally accepted approach

In contrast to the diverse perspectives on whether religion should be incorporated into a PA program, women universally endorsed incorporating concepts of spirituality (i.e., words encouraging connectedness to a higher power, meditation, mind–body activities). Overall, participants emphasized that spirituality was a common ground among AA women, regardless of religious affiliation. One participant illustrated this with the following (Focus Group #1, Participant 4), *“I’m more spiritual than religious now. So for me, maybe having some aspects of the Bible in there would be offensive to me, but I would be receptive to some type of meditation, music or something like that…or even words of affirmation, that would be encouraging as well.”* Participants provided examples of how spirituality could be incorporated into a PA program including: (a) focusing intervention messages to describe how PA can renew one’s mind and sense of purpose, (b) emphasizing that one’s body is their temple and that PA is a self-care activity for health, (c) encouraging women to reflect on how PA can help them align with their spiritual purpose, and (d) including words of affirmation related to PA and one’s spiritual self.

### Discussion

This results of this study explicate how religion and spirituality can be incorporated into a community-based PA program for AA women implemented outside of faith-based or faith-placed setting. A major finding was that spirituality was an acceptable and motivational concept to include in a PA program, regardless of the participant’s religious beliefs. Conversely, religion, while desirable among some women, was considered irrelevant and/or inappropriate by others within the design of a PA program.

Our findings may reflect a changing religious landscape in the U.S. While AAs remain one of the most religious demographic groups in the U.S. [27], recent data indicate younger generations (i.e., Millennials and Generation X) are less religious than their older generation counterparts [28]. The mean age of participants in our study (i.e., 38 years) places our sample in these “less religious” generational cohorts. While interest in formal religion among younger generations may be waning, there appears to be an increase in the number of Americans who associate themselves with being spiritual [28], as data from the Pew Religious Landscape Survey show the number of all Americans who report feeling a “deep sense of spiritual peace and well-being” increased from 52 to 59% between 2007 and 2014 [29]. Although these data on spirituality are not race specific, they do provide some context to our qualitative findings.

Despite some women opposing the use of religion in a community-based PA program, many women endorsed it. These findings highlight that faith-placed and faith-based PA programs have contemporary relevance among many AA women. However, future studies are needed to further explore the efficacy of faith-based and faith-placed approaches to increase PA among AA women. In particular, there is a need for in-depth preliminary work that includes knowledge and sensitivity to generational contextual differences among AA women.

The information gained from this report has implications for both research and practice. First, our data show that incorporating aspects of religion into PA programs for AA women of a homogenous religious faith through faith-based or faith-placed settings may help enhance the salience, uptake, and behavioral targets of the program. Second, when designing PA programs for AA women who do not self-identify as religious or for a diverse group of AA women who may or may not be religious, researchers should avoid mentions of specific religious concepts. Instead, they should consider designing intervention messages to include notions of spirituality or excluding mentions of religion and spirituality altogether.

## Limitations

Limitations of our study include a sample comprised of predominantly highly educated AA women residing in a single in a Southwestern U.S. metropolitan area, truncating generalization to AA women with lower education levels or residing in other regions of the U.S. Despite these limitations, findings provide a basis for future research to explore these topics in a more diverse group of AA women. A strength of our study was that we explored PA design strategies that have not been studied in AA women in recent years. Given that sociocultural norms evolve overtime, these results provide preliminary data on how norms associated with PA, religion, and spirituality may be evolving in the AA community. Future studies are warranted to confirm and expand on our findings.

## Abbreviations

AA: African American
PA: physical activity
U.S.: United States

## References

[1] Centers for Disease Control and Prevention. National Center for Health Statistics, Health Data Interactive. 2015. http://www.cdc.gov/nchs/hdi.htm. Accessed 1 Aug 2015.

[2] Ogden CL, Carroll MD, Flegal KM (2014). Prevalence of obesity in the United States. JAMA.

[3] Mozaffarian D, Benjamin EJ, Go AS (2016). Heart disease and stroke statistics-2016 update: a report from the American Heart Association. Circulation.

[4] Centers for Disease Control and Prevention. Age-adjusted rates of diagnosed diabetes per 100 civilian, non-institutionalized population, by Hispanic origin and sex, United States, 1997–2014. 2015. http://www.cdc.gov/diabetes/statistics/prev/national/fighispanicthsex.htm. Accessed 19 May 2016.

[5] Giger JN, Appel SJ, Davidhizar R (2008). Church and spirituality in the lives of the African American community. J Transcult Nurs.

[6] Douglas KB, Hopson RE (2000). Understanding the black church: the dynamics of change. J Relig Thought.

[7] Bopp M, Fallon E (2012). A comprehensive review of faith-based physical activity interventions. Am J Lifestyle Med.

[8] Lancaster KJ, Carter-Edwards L, Grilo S (2014). Obesity interventions in African American faith-based organizations: a systematic review. Obes Rev.

[9] Quinn ME, Guion WK (2010). A faith-based and cultural approach to promoting self-efficacy and regular exercise in older African American women. Gerontol Geriatr Educ.

[10] Sternberg Z, Munschauer IIIFE, Carrow SS (2007). Faith-placed cardiovascular health promotion: a framework for contextual and organizational factors underlying program success. Health Educ Res.

[11] Yanek LR, Becker DM, Moy TF (2001). Project joy: faith based cardiovascular health promotion for African American women. Public Health Rep.

[12] Hill PC, Pargament K, Hood JRW (2000). Conceptualizing religion and spirituality: points of commonality, points of departure. J Theor Soc Behav.

[13] Pew Research Center. Religious Landscape Survey. 2017. http://www.pewforum.org/religious-landscape-study/. Accessed 24 Feb 2017.

[14] Kharitonov SA (2012). Religious and spiritual biomarkers in both health and disease. Religions.

[15] Miller WR, Thoresen CE (2003). Spirituality, religion, and health: an emerging research field. Am Psychol.

[16] Schneiders SM (2003). Religion vs. spirituality: a contemporary conundrum. Spiritus..

[17] Joseph RP, Ainsworth BE, Mathis L, Hooker SP, Keller C (2017). Utility of social cognitive theory in intervention design for promoting physical activity among African-American women: a qualitative study. Am J Health Behav.

[18] Joseph RP, Keller C, Adams MA (2016). Validity of two brief physical activity questionnaires with accelerometers among African-American women. Prim Health Care Res Dev..

[19] Joseph RP, Ainsworth BE, Keller C (2015). Barriers to physical activity among African American women: an integrative review of the literature. Women Health.

[20] Joseph RP, Pekmezi D, Allison JJ, Durant NH (2014). Lessons learned from the development and implementation two internet-enhanced culturally relevant physical activity interventions for young overweight African American women. J Nat Black Nur Assoc..

[21] Joseph RP, Keller C, Adams MA (2015). Print versus a culturally-relevant Facebook and text message delivered intervention to promote physical activity in African American women: a randomized pilot trial. BMC Womens Health.

[22] Joseph RP, Keller C, Affuso O (2016). Designing culturally relevant physical activity programs for African-American women: a framework for intervention development. J Racial Ethn Health Disparities.

[23] Joseph RP, Pekmezi D, Dutton GR (2014). Results of a culturally adapted internet-enhanced physical activity pilot intervention for overweight and obese young adult African American women. J Transcult Nurs.

[24] Joseph RP, Pekmezi DW, Lewis T (2013). Physical activity and Social Cognitive Theory outcomes of an internet-enhanced physical activity intervention for African American female college students. J Health Disparities Res Pract.

[25] Elo S, Kyngas H (2008). The qualitative content analysis process. J Adv Nurs.

[26] O’Reilly M, Parker N (2012). ‘Unsatisfactory Saturation’: a critical exploration of the notion of saturated sample sizes in qualitative research. Qual Res.

[27] Pew Research Center. Racial and ethnic composition, religious landscape study. 2017. http://www.pewforum.org/religious-landscape-study/racial-and-ethnic-composition/. Accessed 24 Feb 2017.

[28] Aplper BA. Millennials are less religious than older Americans, but just as spiritual. 2015. http://www.pewresearch.org/fact-tank/2015/11/23/millennials-are-less-religious-than-older-americans-but-just-as-spiritual/. Accessed 24 Feb 2017.

[29] Masci DL, Lipka M. Americans may be getting less religious, but feelings of spirituality are on the rise. 2016; http://www.pewresearch.org/fact-tank/2016/01/21/americans-spirituality/. Accessed 24 Feb 2017.

